# A Fish-Derived Protein Hydrolysate Induces Postprandial Aminoacidaemia and Skeletal Muscle Anabolism in an In Vitro Cell Model Using Ex Vivo Human Serum

**DOI:** 10.3390/nu13020647

**Published:** 2021-02-17

**Authors:** Matthew J. Lees, David Nolan, Miryam Amigo-Benavent, Conor J. Raleigh, Neda Khatib, Pádraigín Harnedy-Rothwell, Richard J. FitzGerald, Brendan Egan, Brian P. Carson

**Affiliations:** 1 Department of Physical Education and Sport Sciences, Faculty of Education and Health Sciences, University of Limerick, V94 T9PX Limerick, Ireland; matthew.lees@ul.ie (M.J.L.); conor.raleigh@ul.ie (C.J.R.); 2 School of Health and Human Performance, Dublin City University, D09 V209 Dublin, Ireland; david.nolan@dcu.ie (D.N.); brendan.egan@dcu.ie (B.E.); 3 Health Research Institute, University of Limerick, V94 T9PX Limerick, Ireland; miryam.amigobenavent@ul.ie; 4 Department of Biological Sciences, University of Limerick, V94 T9PX Limerick, Ireland; neda.khatib@ul.ie (N.K.); padraigin.harnedy@ul.ie (P.H.-R.); dick.fitzgerald@ul.ie (R.J.F.)

**Keywords:** ageing, blue whiting, hypertrophy, leucine, mTORC1, muscle protein synthesis

## Abstract

Fish-derived proteins, particularly fish protein hydrolysates (FPH), offer potential as high-quality sources of dietary protein, whilst enhancing economic and environmental sustainability. This study investigated the impact of a blue whiting-derived protein hydrolysate (BWPH) on aminoacidaemia in vivo and skeletal muscle anabolism in vitro compared with whey protein isolate (WPI) and an isonitrogenous, non-essential amino acid (NEAA) control (0.33 g·kg^−1^·body mass^−1^) in an ex vivo, in vitro experimental design. Blood was obtained from seven healthy older adults (two males, five females; age: 72 ± 5 years, body mass index: 24.9 ± 1.6 kg·m^2^) in three separate trials in a randomised, counterbalanced, double-blind design. C2C12 myotubes were treated with ex vivo human serum-conditioned media (20%) for 4 h. Anabolic signalling (phosphorylation of mTOR, p70S6K, and 4E-BP1) and puromycin incorporation were determined by immunoblotting. Although BWPH and WPI both induced postprandial essential aminoacidaemia in older adults above the NEAA control, peak and area under the curve (AUC) leucine and essential amino acids were more pronounced following WPI ingestion. Insulin was elevated above baseline in WPI and BWPH only, a finding reinforced by higher peak and AUC values compared with NEAA. Muscle protein synthesis, as measured by puromycin incorporation, was greater after incubation with WPI-fed serum compared with fasted serum (*P* = 0.042), and delta change was greater in WPI (*P* = 0.028) and BWPH (*P* = 0.030) compared with NEAA. Myotube hypertrophy was greater in WPI and BWPH compared with NEAA (both *P* = 0.045), but was similar between bioactive conditions (*P* = 0.853). Taken together, these preliminary findings demonstrate the anabolic potential of BWPH in vivo and ex vivo, thus providing justification for larger studies in older adults using gold-standard measures of acute and chronic MPS in vivo.

## 1. Introduction

The decline in muscle strength, quality, and physical function with age, otherwise known as sarcopenia [1], is associated with physical disability, an increased risk of falls, decreased quality of life, hospitalisation, and mortality [2,3,4,5,6]. Dietary protein is recognised as an important strategy to manage and prevent sarcopenia, offering substantial therapeutic benefits when applied synergistically with resistance exercise [7,8,9,10]. However, 31–50% of non-institutionalised older adults fail to meet the Recommended Daily Allowance (RDA) for protein of 0.8 g·kg^−1^·body mass^−1^·day^−1^ [11], despite the fact that several authors have advocated intakes in excess of 1.0 g·kg^−1^·body mass^−1^·day^−1^ for this population [12,13,14,15]. Although older people are less responsive to a low-dose amino acid (AA) bolus, such “anabolic resistance” may be overcome with greater protein or essential amino acid (EAA) provision [12,16,17].

The acute postprandial aminoacidaemia observed following the ingestion of dietary protein is a strong driver of muscle protein synthesis (MPS), the amplitude and duration of which is greatly influenced by the type of protein consumed [18,19,20]. In addition, the age of the individual, AA profile of the protein source, its digestion and absorption kinetics, and time course of ingestion all modulate postprandial MPS, and induce varying patterns of aminoacidaemia [21,22,23,24,25,26]. For instance, whey protein hydrolysate (WPH) induces a more pronounced aminoacidaemia than micellar casein [27], free AAs [28], or soy protein isolate [27,29], and is associated with a more robust stimulation of MPS than these other sources [27,29]. This variation is likely due to differences in the speed of digestion (i.e., fast vs. slow [27]), absorption (i.e., the amount of free AAs, peptide chain length [28]), and/or leucine content between protein sources.

Fish-derived protein hydrolysates (FPH) may hold promise as functional foods to promote healthy ageing, metabolic health, and benefit skeletal muscle metabolism in humans [30], whilst improving economic and environmental efficiency [31,32]. Cordeiro et al. [33] reported rapid and robust post-exercise aminoacidaemia in young individuals following consumption of 0.25 g·kg^−1^·body mass^−1^ of a Nile tilapia-derived FPH—a response that was similar to WPH. This suggests that FPH might represent a useful and efficient alternative for the promotion of skeletal muscle anabolism [30]. Nonetheless, the impact of FPH on amino acid kinetics and anabolic potential remains to be determined in older adults.

Recent work in our laboratory has developed an in vitro murine model to evaluate the intracellular anabolic signalling and MPS responses to WPH and its derivatives using ex vivo human serum in myotubes [34,35,36]. The conditioning of culture medium with ex vivo human serum from diverse populations represents a potentially more physiologically relevant model for studying MPS regulation in skeletal muscle cells over traditional cell culture platforms. Our model also presents an attractive “proof of principle” alternative to established methods of MPS determination using stable isotope tracers [37,38,39] that can be costly, invasive, necessitate multiple skeletal muscle biopsies, and pose technical challenges [34].

Therefore, the principal aims of the present study were to (a) investigate the amino acid uptake kinetics of a blue whiting-derived FPH compared with whey protein isolate (WPI) and an isonitrogenous non-essential amino acid (NEAA) control, and (b) characterise the effect of media conditioned with postabsorptive and postprandial serum from healthy older adults on intracellular signaling, MPS, and hypertrophy in C2C12 myotubes.

## 2. Materials and Methods 

### 2.1. Participants and Ethical Approval

The study was approved by the Local Research Ethics Committee at Dublin City University (DCUREC2019/085) and carried out in accordance with the guidelines set forth in the Declaration of Helsinki (1964). Seven (*n* = 7; two males, five females) older adults (age: 72 ± 5 years; height: 1.70 ± 0.09 m; body mass: 71.7 ± 7.5 kg; body mass index: 24.9 ± 1.6 kg·m^2^) defined as medically stable [40] participated in the study after providing written informed consent.

### 2.2. Study Design

Participants reported to the laboratory on three occasions following an overnight fast (>10 h), having refrained from exercise for at least 24 h prior to arrival. All visits were separated by a minimum of seven days. A trained phlebotomist inserted a cannula (21G) into an antecubital vein, and a blood sample was collected at baseline (*t* = 0 min) in S-Monovette^®^ serum separator tubes (SST; Sarstedt, Wexford, Ireland). Subsequently, participants consumed 0.33 g protein·kg^−1^·body mass^−1^ of FPH, WPI, or a non-bioactive isonitrogenous NEAA control dissolved in 400 mL water (7.6% *w/v*; AA composition of test drinks provided in Table 1) in a randomised, double-blind, and counterbalanced manner. Participants were dosed on a per-protein basis, with conditions matched for total AAs and nitrogen. Upon commencing ingestion of a given drink, participants had five minutes to complete ingestion. Blood samples were collected at baseline (*t* = 0 min) and 15, 30, 45, 60, 90, 120, 150, and 180 min after commencing ingestion. After clotting at room temperature for 20 min, samples were moved to an ice slurry for storage until the end of each trial, after which they were centrifuged (15 min × 3000× *g* at 4 °C). Separated serum was stored at −80 °C until subsequent analysis or experimentation. Serum samples from *t* = 0 min and *t* = 60 min were designated as FASTED and FED, respectively, for subsequent use in the below-described cell culture experiments.

### 2.3. Generation of Blue Whiting Protein Hydrolysate

The BWPH was generated using a proprietary process at Bio-Marine Ingredients Ireland Ltd. (Lough Egish Food Park, Castleblaney, Co. Monaghan, Ireland). The protein-equivalent content of the sample, as determined by Harnedy et al. [41], was found to be 87.79 ± 0.63% (*w/w*; moisture: 4.19% (*w/w*); ash: 6.90% (*w/w*)). The degree of hydrolysis, as determined by Le Maux et al. [42], was found to be 35.40 ± 1.81%, and 78.78% of components, as determined by Spellman et al. [43], were identified as being <1 kDa.

### 2.4. Measurement of Amino Acids and Insulin Concentrations

Serum AAs were analysed using the Agilent 1200 reversed-phase ultra-performance liquid chromatography (RP-UPLC) system (Agilent Technologies Inc., Santa Clara, CA, USA) equipped with an Agilent 1260 binary pump and a G1367C automated liquid handling system, with AA separation, data acquisition, and quantitative analysis all carried out as previously described by our group [34,35,44]. Insulin concentrations were determined using a commercially available, enzyme-linked immunosorbent assay (ELISA) kit (10-113-01; Mercodia, Sweden). In addition to time-series data, peak concentration (C_max_) and time to peak concentration (T_max_) were calculated for total AAs, NEAAs, EAAs, branched-chain amino acids (BCAAs), leucine, and insulin. Area under the curve (AUC) was determined for all serum variables using the trapezoidal method. Time-series values for individual AAs are provided as Supplementary Data.

### 2.5. Mammalian Cell Culture

The murine cell line C2C12 (91031101; Sigma-Aldrich, Gillingham, United Kingdom) was used to perform all in vitro experiments. Cells were cultured and sub-cultured in DMEM (D6429; Sigma-Aldrich) supplemented with 10% (*v/v*) fetal bovine serum (F7524, Sigma-Aldrich), 1% (*v/v*) penicillin/streptomycin solution (P0781; Sigma-Aldrich), and 1 mM L-glutamine (G7513; Sigma-Aldrich) at 5% CO_2_ in a humidified atmosphere at 37 °C. At 70% confluence, cells were differentiated (up to seven days) in DMEM supplemented with 2% horse serum (H1270; Sigma-Aldrich) as previously described [34,45]. Prior to treatment with human serum-conditioned media, fully differentiated myotubes were nutrient-deprived in AA and serum-free DMEM (D9807-10; United States Biological, Salem, Massachusetts, USA) supplemented with 1 mM sodium pyruvate (11360-070; Thermo Fisher, Dublin, Ireland), 1% (*v/v*) penicillin/streptomycin solution, 1 mM L-glutamine, 6 mM D-glucose, and 34 mM NaCl (all Sigma-Aldrich, Gillingham, United Kingdom; pH adjusted to 7.3).

### 2.6. Muscle Protein Synthesis

The surface-sensing of translation (SUnSET) technique was used to measure muscle protein synthesis in C2C12 myotubes [46,47], after treatment with media conditioned by ex vivo human serum, as previously described [34,35]. Differentiated and mature C2C12 myotubes were nutrient-deprived in AA and serum-free DMEM for 1 h, after which they were treated with media containing 20% human serum and 1 µM puromycin (Merck-Millipore Limited, Carrigtwohill, Co. Cork, Ireland) for 4 h, after which protein lysates were obtained. The nutrient deprivation time, puromycin concentration, treatment duration, and percentage of human serum were optimised in previously published work from our laboratory [34]. 

### 2.7. Immunoblotting

Protein lysates were obtained by placing cells on ice, aspirating the medium, and washing three times with cold phosphate-buffered saline (C-40232; PromoCell, Brennan & Co., Stillorgan, Co. Dublin, Ireland). Lysis buffer was added to each well, then scraped and collected in microcentrifuge tubes, as previously described [34,35]. Lysates were subsequently placed on ice for 20 min prior to centrifugation at 130× *g* for 15 min at 4 °C. Total protein content was determined from the supernatant in triplicate using the Bradford assay [48].

Equal amounts of protein were diluted in 4X Laemmli buffer and denatured (95 °C for 5 min) and loaded onto 4–15% linear gradient SDS-PAGE precast gels (Mini-Protean TGX Stain-Free, 456-8083, Bio-Rad) alongside a protein ladder (26619, 26625; Thermo Fisher, Dublin, Ireland). Following separation, total protein (loading control) in each lane was determined using stain-free, UV-induced fluorescence to activate tryptophan residues on each gel (UVI-TEC Cambridge Imaging System, UVITEC, Cambridge, United Kingdom). Proteins were electroblotted onto a 0.2 µm nitrocellulose membrane using the semi-dry transfer technique (Trans-Blot^®^ Turbo^TM^; Bio-Rad). Membranes were then blocked for 1 h at room temperature in Tris-buffered saline with 0.05% (*v*/*v*) Tween 20 (TBST) containing 5% bovine serum albumin (BSA). Subsequently, membranes were washed four times with TBST and incubated with primary antibodies at 4 °C overnight. All primary antibodies for intracellular signaling (Cell Signaling, Bioke, Leiden, The Netherlands) were diluted at 1:1000 in TBST and 5% BSA: total mechanistic Target of Rapamycin (mTOR) (#2972S), phospho-mTOR^Ser2448^ (#5536S), total Eukaryotic translation initiation factor 4E (eIF4E)-binding protein 1 (4E-BP1) (#9644S), phospho-4E-BP1^Thr37/46^ (#2855S), total P70 S6 kinase (P70S6K) (#2708S), and phospho-P70S6K^Thr389^ (#9234S). Puromycin was probed using MABE343 anti-puromycin, clone 12D10 mouse monoclonal antibody (Merck Millipore Limited, Carrigtwohill, Co. Cork, Ireland). Following incubation, membranes were washed four times with TBST and incubated with the secondary antibody goat anti-rabbit IgG (#926-32211; IRDye® 800 CV; LI-COR Biosciences UK Limited, Cambridge, UK) for all primary antibodies except for puromycin, which was incubated with goat anti-mouse IgG2a-specific antibody (#926-32351; LI-COR Biosciences UK Limited, Cambridge, UK). After image capture, phospho-specific blots were stripped using 1X stripping buffer (ReBlot Plus Strong Antibody Stripping Solution; 2504, Merck Millipore Ltd.), blocked once again, then reprobed using pan antibodies. 

Whole-lane (i.e., Stain-Free total protein and puromycin) and single-band (i.e., anabolic signalling) densitometry was performed in ImageJ (National Institutes of Health, Baltimore, MD, United States; [49]). Total and phosphoprotein band density was normalised to the Stain-Free total protein lane density obtained from each gel [50] and the phospho-to-total protein ratio was determined. Immunoblot data are expressed as normalised phosphoprotein divided by normalised total protein (phospho/total) in absolute arbitrary units (AU) and as difference scores (delta).

### 2.8. Myotube Thickness

Myotube thickness was assessed using an inverted light microscope (CKX31, Olympus Life Science, Shinjuku, Tokyo, Japan) following 1 h of serum starvation and again following 4 h of incubation with human serum-conditioned media. Images were captured, saved as JPEG files, and analysed using ImageJ (V1.52, National Institutes of Health). Diameter was measured at three locations along the length of each myotube, in at least three different fields, for a minimum of 100 myotubes per treatment condition [45,51].

### 2.9. Statistical Analysis

All statistical procedures were performed using GraphPad Prism v8.3.1 for Windows (GraphPad Software, La Jolla, CA, USA). Data were tested for normality using the Shapiro-Wilk test and visualisation of normality plots. Homogeneity of variance was assessed using Levene’s test. For AA time-series data, anabolic signaling, puromycin incorporation, and myotube hypertrophy variables were analysed using two-way repeated measures analysis of variance (ANOVA) with time and condition (i.e., NEAA, WPI, and BWPH) as factors. Serum C_max_, T_max_, and AUC values were analysed using one-way repeated measures ANOVA, as were delta values for anabolic signaling, MPS, and myotube hypertrophy. In the event that significant main and/or interaction effects were found, post hoc tests were performed using the Holm–Bonferroni adjustment for multiple comparisons. Where appropriate, the magnitude of observed effects was quantified using Cohen’s *d* and interpreted with the following thresholds: <0.2 = trivial, 0.2–0.5 = small, 0.5–0.8 = moderate, and ≥0.8 = large [52]. The level of significance for all analyses was set at 95% (α = 0.05) with data presented as mean ± standard deviation (SD), unless otherwise stated.

## 3. Results

### 3.1. Serum Amino Acids and Insulin

**Figure 1:**
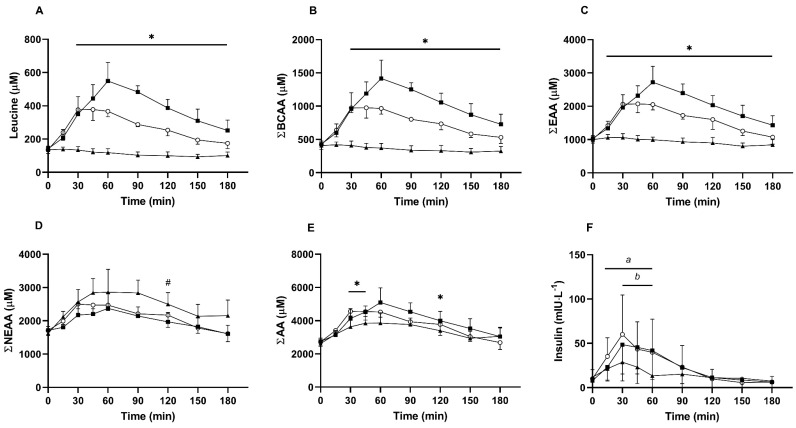
Serum amino acid ((**A**): Leucine, (**B**): Sum Branch Chain Amino Acids (BCAA), (**C**): Sum Essential Amino Acids (EAA), (**D**): Sum Non-Essential Amino Acids (NEAA), (**E**): Sum Total Amino Acids (AA)) and insulin concentrations (**F**) in response to non-essential amino acids (NEAA, ▲), whey protein isolate (WPI, ■), and blue whiting protein hydrolysate (BWPH, ○) in healthy older adults. Data are presented as mean ± SD. * Significant differences between conditions at each time point (see text for further details); ^#^ NEAA significantly higher than WPI; ^a^ significantly different to baseline in BWPH; ^b^ significantly different to baseline in WPI.

Leucine: For time-series data, there was a significant main effect of time, condition, and time × condition interaction (all *P* < 0.001; Figure 1A). Post hoc testing revealed that leucine did not differ at baseline between conditions (all comparisons *P* > 0.700; *d* ≤ 0.40). After 30 min, leucine was significantly elevated in both WPI and BWPH compared with the NEAA control (*d* = 2.87 and *d* = 8.21, respectively), and these differences persisted until the conclusion of the trial (all *P* < 0.05). After 60 min, leucine was significantly higher in WPI compared with BWPH (*d* = 2.23) and remained so until 180 min (all *P* < 0.05). 

Leucine C_max_ differed between groups (*P* < 0.001; see Table 2) with higher values seen in WPI and BWPH compared to NEAA (both *P* < 0.001; *d* = 8.56 and *d* = 7.40, respectively). WPI also demonstrated a higher leucine C_max_ than BWPH (*P* = 0.004; *d* = 3.00). Time to peak leucine concentration (T_max_) also differed between groups (*P* < 0.001), with NEAA occurring earlier than WPI (*P* = 0.002; *d* = 4.10) and BWPH (*P* = 0.015; *d* = 2.26), respectively. Leucine T_max_ in WPI was also later than that following BWPH (*P* = 0.004; *d* = 2.33). Leucine AUC was different between groups (*P* < 0.001) with NEAA smaller than both WPI and BWPH (both *P* < 0.0001; *d* = 11.01 and *d* = 11.00, respectively), and WPI larger than BWPH (*P* < 0.0001; *d* = 4.96).

BCAA: Time-series data for BCAAs (Figure 1B) demonstrated main effects for time, condition, and a time x condition interaction (all *P* < 0.001). Post hoc testing revealed no differences at baseline (all *P* > 0.999; *d* ≤ 0.34). After 30 min, BCAA concentrations were higher in WPI and BWPH compared with NEAA (*d* = 3.08, *d* = 8.01, respectively), and remained so for the duration of the trial (all *P* < 0.05). After 60 min, BCAA concentration was higher in WPI compared with BWPH (*d* = 2.27) and this also persisted for the duration of the trial (all *P* < 0.05). 

BCAA C_max_ differed between groups (*P* < 0.001; Table 2), whereby WPI was higher than BWPH (*P* = 0.003; *d* = 2.94) and NEAA (*P* < 0.001; *d* = 7.52), respectively. BWPH was also higher than NEAA (*P* < 0.001; *d* = 6.53). Time to peak BCAA concentration differed between groups (*P* < 0.001). BCAA T_max_ was earlier in NEAA compared with WPI (*P* = 0.005; *d* = 3.93) and BWPH (*P* = 0.021; *d* = 2.16), and was also earlier in BWPH compared with WPI (*P* = 0.021; *d* = 1.92). A significant effect was found for BCAA AUC (*P* < 0.001), with NEAA smaller than WPI and BWPH (both *P* < 0.001; *d* = 9.38 and *d* = 8.49, respectively). BWPH AUC was smaller than that for WPI (*P* < 0.001; *d* = 4.70).

EAA: For time-series data (Figure 1C), significant main effects were found for time, condition, and the time x condition interaction (all *P* < 0.001). Serum EAA did not differ between groups at baseline (*P* > 0.701; *d* ≤ 0.39), but after 15 min, concentrations were higher in BWPH compared with NEAA (*P* = 0.022; *d* = 3.20). At 30 min, WPI (*P* = 0.020; *d* = 3.15) and BWPH (*P* < 0.001; *d* = 7.55) were both greater than NEAA. In WPI, EAA remained elevated compared with NEAA for the duration of the trial (all *P* < 0.001), whereas BWPH was similar to NEAA by 180 min (*P* = 0.304; *d* = 1.24). WPI and BWPH were similar until 90 min (all *P* > 0.130), after which WPI was consistently higher at 120 and 150 min (all *P* < 0.05; *d* = 1.47 and *d* = 1.82, respectively) but were similar at 180 min (*P* = 0.104; *d* = 1.45). 

EAA C_max_ significantly differed between groups (*P* < 0.001; Table 2). WPI and BWPH were both higher than NEAA (both *P* < 0.001; *d* = 6.78 and *d* = 7.16, respectively) and WPI was higher than BWPH (*P* = 0.012; *d* = 2.47). Time to peak concentration was significantly different between groups (*P* < 0.001). NEAA T_max_ was earlier than that for WPI (*P* = 0.002; *d* = 3.22) and BWPH (*P* = 0.030; *d* = 1.54), whereas BWPH was lower than WPI (*P* = 0.030; *d* = 1.77). EAA AUC significantly differed between groups (*P* < 0.001), with WPI greater than BWPH (*P* < 0.001; *d* = 3.08) and NEAA (*P* < 0.001; *d* = 7.83), respectively. BWPH was also significantly greater than NEAA (*P* < 0.001; *d* = 7.27).

NEAA: For time-series data (Figure 1D), significant main effects were found for time (*P* < 0.001), condition (*P* < 0.001), but not the time x condition interaction (*P* = 0.082). Following post hoc testing, NEAAs did not differ between conditions at baseline (all *P* > 0.287; *d* ≤ 0.79). At 120 min, NEAAs were greater in NEAA compared with WPI (*P* = 0.044; *d* = 1.67), with no other differences observed by time point or condition (*P* > 0.142). 

NEAA C_max_ significantly differed between groups (*P* = 0.019; Table 2), with NEAA higher than WPI (*P* = 0.041; *d* = 1.78) and BWPH (*P* = 0.003; *d* = 1.77), with no difference between WPI and BWPH (*P* = 0.509; *d* = 0.44). NEAA T_max_ did not significantly differ between conditions (*P* = 0.303). NEAA AUC significantly differed between conditions (*P* < 0.001), with NEAA greater than WPI (*P* = 0.006; *d* = 2.26) and BWPH (*P* = 0.006; *d* = 2.14), with no difference between WPI and BWPH (*P* = 0.248; *d* = 0.72). 

AA: For time-series data (Figure 1E), significant main effects were found for time (*P* < 0.001), condition (*P* = 0.004), and the time x condition interaction (*P* = 0.001). Serum AAs did not differ at baseline between conditions (all *P* > 0.095; *d* ≤ 0.66). At 30 and 45 min, AAs were significantly higher in BWPH compared with NEAA (*P* = 0.007 and *P* = 0.028; *d* = 2.78 and *d* = 1.51, respectively) and were similar to WPI (*P* > 0.999); however, WPI did not differ from NEAA at these time points (*P* > 0.999). In WPI, AAs were elevated compared with NEAA at 120 min (*P* = 0.022; *d* = 1.17). 

AA C_max_ significantly differed between groups (*P* = 0.019; Table 2) with NEAA lower than BWPH (*P* = 0.006; *d* = 1.81) and WPI (*P* = 0.015; *d* = 2.19), respectively. Peak AA concentration did not differ between WPI and BWPH, despite comprising a large effect (*P* = 0.179; *d* = 1.01). AA T_max_ was not significantly different between groups (*P* = 0.376). AA AUC significantly differed by condition (*P* = 0.003), with NEAA smaller than BWPH (*P* = 0.034; *d* = 1.54) and WPI (*P* = 0.007; *d* = 2.01), respectively. AA AUC did not differ between WPI and BWPH (*P* = 0.109; *d* = 0.99). Individual amino acid concentrations for all AA following ingestion of NEAA, WPI and BWPH in serum obtained from healthy older adults are shown in Figure S1. 

Insulin: For time-series data (Figure 1F), significant main effects were found for time (*P* < 0.001), condition (*P* = 0.010), and the time x condition interaction (*P* = 0.004). Post hoc testing did not reveal any between-group differences at baseline (all *P* ≥ 0.150) or any other time point (all *P* ≥ 0.105). Within-condition analyses revealed that in WPI, insulin was increased from baseline at 30 min (*P* < 0.001; *d* = 1.64), 45 min (*P* < 0.001; *d* = 1.65), and 60 min (*P* < 0.001; *d* = 1.09), but was similar to baseline after 90 min (all *P* ≥ 0.589; *d* ≤ 1.12). In BWPH, insulin was significantly elevated at 15 min (*P* = 0.006; *d* = 1.52), 30 min (*P* < 0.001; *d* = 1.53), 45 min (*P* < 0.001; *d* = 1.43), and 60 min (*P* < 0.001; *d* = 1.11), but was similar to baseline after 90 min (all *P* ≥ 0.543; *d* ≤ 0.49). By contrast, insulin did not differ from baseline in NEAA at any time point (all *P* ≥ 0.340; *d* ≤ 0.99).

Insulin C_max_ significantly differed between groups (*P* = 0.006; Table 2), with WPI and BWPH higher than NEAA (both *P* = 0.011; *d* = 1.01, *d* = 0.95, respectively). There was no significant difference in peak serum insulin between WPI and BWPH (*P* = 0.833; *d* = 0.05). Time to peak insulin concentration also did not significantly differ between groups (*P* = 0.695). Insulin AUC differed between groups (*P* = 0.007), with WPI (*P* = 0.016; *d* = 0.54) and BWPH (*P* = 0.010; *d* = 0.54) both greater than NEAA. There was no difference in insulin AUC between WPI and BWPH (*P* = 0.652; *d* = 0.06). 

### 3.2. Intracellular Signaling

**Figure 2:**
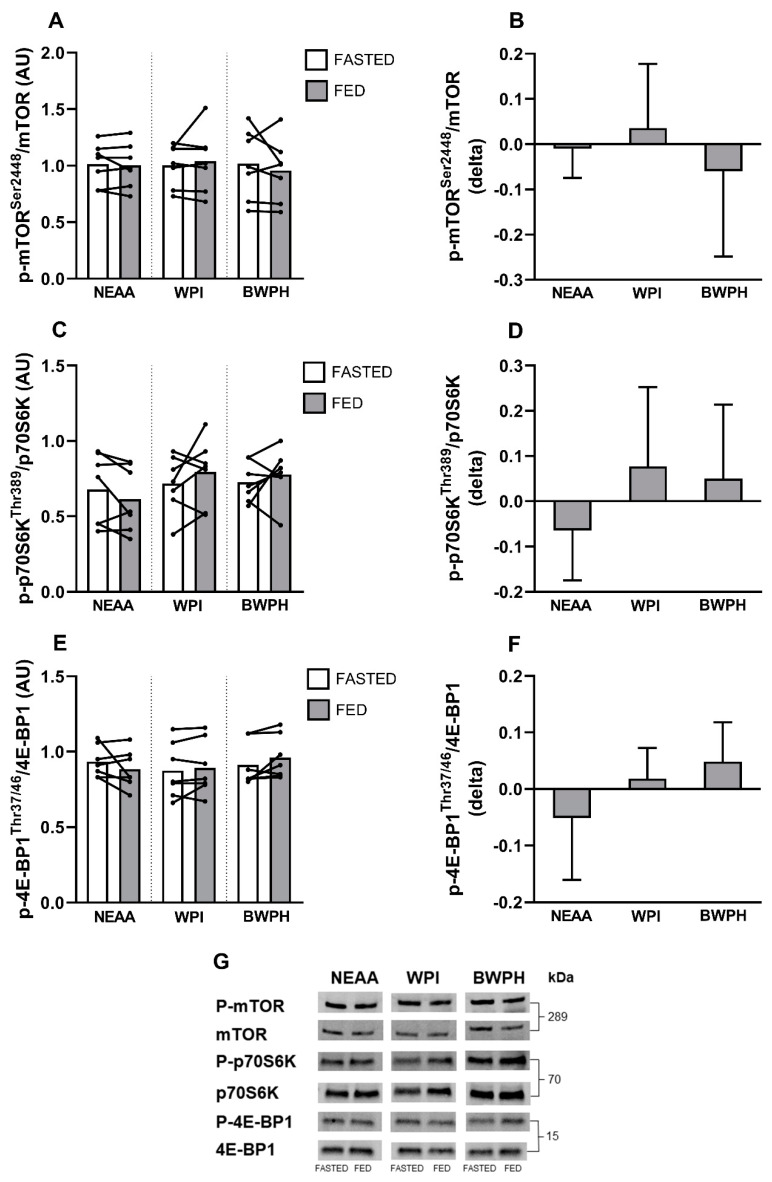
Phosphorylation of mTOR^Ser2448^ (**A**), p70S6K^Thr389^ (**C**), and 4E-BP1^Thr37/46^ (**E**) in response to 1 h incubation with FASTED or FED (NEAA, WPI, and BWPH) human-conditioned serum. Densitometric analyses are reported in absolute terms (arbitrary units, AU) and difference scores (delta; (**B**,**D**,**F**), respectively). (**G**) Representative immunoblots for each total and phosphoprotein.

As demonstrated previously, fasted serum is sufficiently bioactive to induce phosphorylation of mTOR^Ser2448^, p70S6K^Thr389^, and 4E-BP1^Thr37/46^ with little to no additional effect of feeding [34]. Therefore, as expected, ANOVA found no significant effects for time (*P* = 0.775), condition (*P* = 0.671), or the time x condition interaction (*P* = 0.366) for mTOR^Ser2448^ signaling (Figure 2A; representative immunoblots provided in Figure 2G). When expressed as difference scores (Figure 2B), no effect for condition was found (*P* = 0.364).

Similarly, there were no effects of time (*P* = 0.524), condition (*P* = 0.165), or the time x condition interaction (*P* = 0.250) in the phosphorylation of p70S6K^Thr389^ (Figure 2C). When expressed as difference scores (Figure 2D), there was also no significant effect of condition (*P* = 0.250). 

In line with this, there were no effects of time (*P* = 0.741), condition (*P* = 0.379), or the time x condition interaction (*P* = 0.140) on the phosphorylation of 4E-BP1^Thr37/46^ (Figure 2E). When expressed as difference scores (Figure 2F), no significant effect for condition was found (*P* = 0.140).

### 3.3. Muscle Protein Synthesis

**Figure 3:**
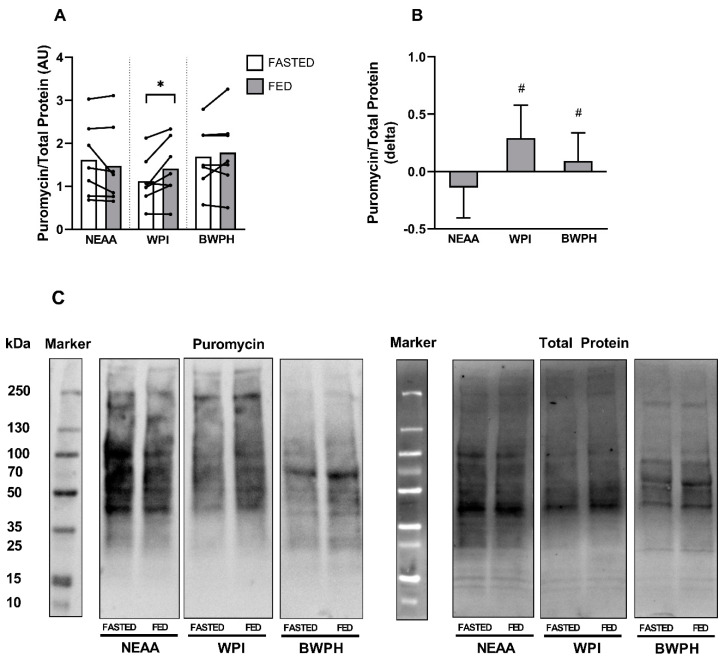
Puromycin incorporation in response to treatment with ex vivo (FASTED and FED) human-conditioned serum (*n* = 7). (**A**) Densitometric analysis of puromycin incorporation in the FASTED and FED state in response to non-essential amino acids (NEAA), whey protein isolate (WPI), and blue whiting protein hydrolysate (BWPH). (**B**) Puromycin incorporation expressed as difference scores (delta). (**C**) Representative immunoblot of puromycin incorporation and total protein (loading control). Data reported as mean ± SD. * Significantly different between FASTED and FED serum; ^#^ significantly different compared with NEAA.

Puromycin incorporation in response to FASTED and FED serum is shown in Figure 3A,C. Two-way repeated measures ANOVA revealed a significant main effect of condition (*P* = 0.025) and a time x condition interaction (*P* = 0.034), but no main effect of time (*P* = 0.204). Post hoc testing indicated that MPS was different between FASTED and FED serum in WPI (*P* = 0.042; *d* = 0.45), but not NEAA or BWPH (both *P* = 0.385; *d* = 0.16 and *d* = 0.12, respectively). When data were expressed as difference scores (Figure 3B), one-way repeated measures ANOVA found a significant effect (*P* = 0.034). Post hoc testing revealed statistically significant effects between NEAA and WPI (*P* = 0.028; *d* = 1.56), and NEAA and BWPH (*P* = 0.030; *d* = 0.90), with no difference between WPI and BWPH (*P* = 0.097; *d* = 0.74). 

### 3.4. Myotube Thickness

**Figure 4:**
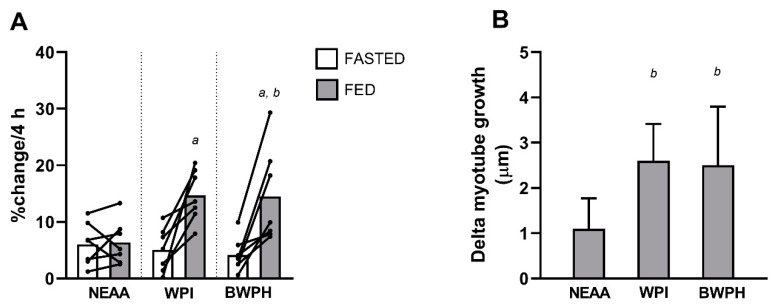
(**A**) Percentage change in myotube thickness following 4 h incubation with FASTED or FED (non-essential amino acid, NEAA; whey protein isolate, WPI; blue whiting protein hydrolysate, BWPH) human-conditioned serum. (**B**) Delta myotube growth (µm) in each condition. ^a^ Significantly different from FASTED, ^b^ significantly different from NEAA-fed.

The percentage change in myotube thickness induced by FASTED and FED serum over the 4 h incubation is shown in Figure 4A. Significant effects of time (*P* = 0.003) and the time x condition interaction (*P* = 0.025) were found, but not condition (*P* = 0.233). Post hoc testing revealed no significant difference in hypertrophy between FASTED and FED serum in NEAA (*P* = 0.999; *d* = 0.08), but significant differences were observed in the WPI (*P* = 0.028; *d* = 2.25) and BWPH (*P* = 0.032; *d* = 1.63) conditions. No differences in myotube hypertrophy were observed between any of the fasted conditions (all *P* > 0.113; *d* = 0.26–0.55). BPWH-fed serum demonstrated greater hypertrophy compared with NEAA (*P* = 0.028; *d* = 1.24), but did not significantly differ from WPI (*P* = 0.999; *d* = 0.03). WPI did not significantly differ from NEAA, but nevertheless demonstrated a large effect (*P* = 0.081; *d* = 1.95). When expressed as delta values (Figure 4B), ANOVA revealed significant differences between conditions (*P* = 0.026). Post hoc testing indicated that WPI and BWPH were both significantly greater than NEAA (both *P* = 0.045; *d* = 2.00 and *d* = 1.34, respectively), with no difference between WPI and BWPH (*P* = 0.853; *d* = 0.09).

## 4. Discussion

To our knowledge, this study is the first to characterise the aminoacidaemic response to a BWPH in older adults compared with both positive (i.e., WPI) and negative (i.e., NEAA) controls. Differences were observed between WPI and BWPH for postprandial aminoacidaemia (i.e., leucine, BCAAs, and EAAs) despite ingestion of isonitrogenous quantities of the proteins. However, the anabolic potential of the proteins studied in vitro using ex vivo postprandial serum was observed to be similar for WPI and BWPH.

The rapid postprandial elevation of EAA concentrations—and leucine in particular—is a key driver of MPS [23,53]. However, the nature of the aminoacidaemia is determined to a great extent by the type, source, and AA profile of the protein source consumed [19,21,26,28]. In the present study, we observed acute postprandial aminoacidaemia (leucine, BCAAs, and EAAs) in response to BWPH, demonstrating comparable bioavailability of EAAs with WPI; however, aminoacidemia was more pronounced following ingestion of WPI due to greater starting EAAs (36% higher in WPI compared to BWPH, Table 1) when matched for nitrogen. Peak leucine concentration was higher following WPI compared to BWPH (~45%), as were peak BCAA (~46%) and EAA (~32%) concentrations. Similarly, the 3 h AUC values for EAAs were greater for WPI compared with BWPH. These divergent responses were likely a consequence of the relatively lower leucine and EAA content of the BWPH (42% less leucine and 36% less EAA, Table 1) than WPI. As such, the rise in essential aminoacidaemia in BWPH was comparable to WPI up to 30 min, suggesting similar digestibility/absorption, but the higher EAA content in WPI resulted in greater peak and AUC EAA concentrations in this condition. Nevertheless, the increase in serum leucine in both test conditions was in excess of 80 µmol·L^−1^, which is the threshold purported to be necessary for MPS activation in vivo [54,55], suggesting that aminoacidaemia induced by BWPH would be sufficient to stimulate MPS in vivo. Our findings are in contrast to those of Cordeiro et al. [33], who found similar post-exercise aminoacidaemia following ingestion of a Nile tilapia-derived FPH and a WPH in young individuals. However, it must be appreciated that the dose consumed was smaller than in the present study (0.25 g·kg^−1^·body mass^−1^), and the AA profile of a given FPH can differ depending on its source species [30]. Irrespective of these discrepancies, the NEAA beverage did not induce a change in serum EAA concentrations from fasting levels, thus further supporting the efficacy of this formulation as a non-bioactive isonitrogenous control. 

A similar insulinotropic effect was observed in WPI and BWPH, with no difference in peak and AUC concentrations between conditions. Insulinaemia was induced 15 min earlier in BWPH compared with WPI; however, concentrations were similarly elevated above baseline at the 60 min time point. It is important to note that we compared an intact protein source (WPI) to a hydrolysed formulation (BWPH) with ~79% of components < 1 kDa in size. The composition of free AAs, peptides, and polypeptides of a protein source is known to affect the route of absorption (i.e., AA transporters or peptide transporters) [28,56]. Therefore, the fact that the BWPH already contained free AAs and short peptides may partly explain the earlier insulinaemia, due to the uptake of small components across the gastrointestinal membrane. Nevertheless, concentrations were similar to baseline in both conditions beyond 90 min. Despite the elevated EAAs in WPI, the lack of a difference in peak and AUC insulin concentrations may have been underpinned by the elevated concentrations of key insulinotropic AAs in BWPH, such as arginine (see Supplemental Data). The insulinotropic action of protein ingestion is known to be “permissive” for MPS, as opposed to modulatory [57,58,59]. Rather, insulin appears to regulate processes that inhibit muscle protein breakdown in an AA-independent manner [59,60]. The lack of appreciable change in serum insulin, allied to EAA concentrations that did not deviate from fasted concentrations, further reinforces the utility of the NEAA formulation as a non-bioactive control. We suggest that it is principally the difference in EAA concentrations between conditions that determined the differential in MPS and myotube diameter observed here. 

We treated C2C12 myotubes with human serum-conditioned media, using our previously published experimental model [34,35,36] to investigate the potential of BWPH to activate MPS. None of the experimental conditions stimulated the phosphorylation of mTOR^Ser2448^ or its downstream signaling proteins p70S6K^Thr389^ and 4E-BP1^Thr37/46^ beyond the level of FASTED serum. This finding agrees with our previous study in which we demonstrated that fasted serum stimulates phosphorylation of all three kinases [34]. These data indicate that all conditions, including BWPH-fed, were sufficient to “switch on” or activate the mTORC1 and downstream MPS signaling pathway that is a prerequisite for the EAA-induced stimulation of MPS in humans [61]. Having confirmation of phosphorylation of the mTOR pathway, we investigated the incorporation of puromycin into proteins as a measure of MPS using the SUnSET technique [46,47]. Our non-bioactive control condition induced no increase in puromycin incorporation. Only our bioactive control (WPI-fed serum) stimulated a significant increase in puromycin incorporation when compared to FASTED serum. Though BWPH did not significantly increase puromycin incorporation above the fasted condition, this was likely due to sample size, as a large effect size was observed between BWPH-fed and fasted conditions. When expressed as difference scores, puromycin incorporation was significantly elevated in both WPI and BWPH conditions, with large effect sizes observed. Taken together, these data suggest that both WPI and BWPH are bioactive for the stimulation of MPS. To further establish the anabolic potential of BWPH, we analysed the impact of fed serum on skeletal muscle myotube thickness as a measure of myofibrillar hypertrophy. Despite the small anabolic signaling responses in the test conditions, myotube hypertrophy was found after incubation with WPI-fed and BWPH-fed serum (both ~15%), thereby supporting the anabolic effects of both supplements when allied to the findings from SUnSET analyses. These effects were likely consequent to, and follow the pattern of, the concentration of circulating drivers of anabolism (i.e., EAAs) reported here.

### Limitations

It is important to note that the sample size may have contributed to a lack of statistical power for some measures, as indicated by the large effect sizes observed in the absence of statistically significant findings. Though a greater *n* was planned at the outset, this randomised crossover study was halted by restrictions associated with the COVID-19 pandemic (March–July 2020). Due to the at-risk population concerned, and the end of the funding term, recruitment was discontinued. This resulted in an imbalance in the sex of the participants; however, we have no reason to believe this further confounds the results at this time. Although large variability was evident and the results should be interpreted with caution, the sample size used in the present study is consistent with previous investigations using this model [34,35,36]. It is also important to note that, even though postprandial serum from older adults was used for the in vitro experiments, the markers of anabolism reported in this study are confined to an in vitro model and may not necessarily translate to in vivo settings. Nevertheless, we assert that the present evidence provides a solid rationale to conduct further human investigations to confirm or deny this supposition.

## 5. Conclusions

The findings of the present study indicate that BWPH induces robust essential aminoacidaemia in older adults, albeit not to the same extent as WPI, due to the divergent AA profiles of the respective test samples. Using our cell-based experimental model, we also provided preliminary evidence of the effects of BWPH on MPS and myotube growth in vitro. These findings provide a rationale for further investigations in older adults to more cogently reveal the impact of BWPH on skeletal muscle metabolism in vivo. Such studies may utilise existing measures of acute and chronic MPS, as well as whole-body net protein balance in conjunction with molecular transducers of skeletal muscle anabolism. Based on current evidence, this BWPH may offer potential as an alternative source of readily bioavailable protein to support skeletal muscle health and anabolism in older people. 

## Figures and Tables

**Table 1:** Amino acid composition of the negative control (NEAA), positive control (WPI), and BWPH beverages.

Amino Acid (g·100 g Powder)	NEAA	WPI	BWPH
***Essential***			
Leucine	0.00	9.87	5.78
Isoleucine	0.00	7.26	3.35
Valine	0.00	6.62	3.89
Lysine	0.00	9.75	7.44
Histidine	0.00	1.54	1.53
Methionine	0.00	2.15	1.94
Phenylalanine	0.00	2.80	2.87
Threonine	0.00	7.07	3.70
Tryptophan	0.00	1.60	0.65
***Non-essential***			
Alanine	13.90	5.25	5.41
Arginine	0.00	1.89	5.94
Aspartic Acid	20.70	10.60	8.56
Cysteine	0.00	2.37	0.61
Glutamic Acid	28.70	18.10	12.50
Glycine	14.20	1.51	5.52
Proline	8.02	6.04	3.41
Serine	10.60	4.96	4.10
Tyrosine	7.40	2.67	3.23
**ΣBCAAs**	0.00	23.75	13.02
**ΣEAAs**	0.00	48.66	31.15
**ΣNEAAs**	103.52	53.39	49.28
**ΣAAs**	103.52	102.05	80.43

*Abbreviations*: AAs, total amino acids; BCAAs, branched-chain amino acids; BWPH, blue whiting protein hydrolysate; EAAs, essential amino acids; NEAAs, non-essential amino acids; WPI, whey protein isolate.

**Table 2:** Peak concentration, time to peak concentration, and area under the curve for serum amino acids and insulin following each experimental condition in healthy older adults (*n* = 7). Data presented as mean ± SD.

Variable	NEAA	WPI	BWPH
***Leucine***			
C_max_ (µM)	141 ± 15	580 ± 71 ^a^	399 ± 47 ^a, b^
T_max_ (min)	15 ± 12	73 ± 16 ^a^	41 ± 11 ^a, b^
AUC (µM·3 h)	19,977 ± 3376	67,614 ± 5105 ^a^	48,848 ± 1546 ^a, b^
***ΣBCAAs***			
C_max_ (µM)	432 ± 51	1488 ± 192 ^a^	1021 ± 117 ^a, b^
T_max_ (min)	19 ± 11	73 ± 16 ^a^	45 ± 13 ^a, b^
AUC (µM·3 h)	63,674 ± 11,134	182,551 ± 14,036 ^a^	134,220 ± 3777 ^a, b^
***ΣEAAs***			
C_max_ (µM)	1092 ± 113	2855 ± 350 ^a^	2168 ± 180 ^a, b^
T_max_ (min)	28 ± 10	69 ± 15 ^a^	45 ± 12 ^a, b^
AUC (µM·3 h)	168,288 ± 19,649	358,693 ± 28,225 ^a^	290,640 ± 13,419 ^a, b^
***ΣNEAAs***			
C_max_ (µM)	3154 ± 344	2519 ± 369 ^a^	2651 ± 210^a^
T_max_ (min)	84 ± 49	60 ± 15	58 ± 31
AUC (µM·3 h)	446,214 ± 38,004	361,362 ± 37,120 ^a^	382,311 ± 18,305 ^a^
***ΣAAs***			
C_max_ (µM)	4154 ± 347	5354 ± 693 ^a^	4795 ± 362 ^a^
T_max_ (min)	81 ± 50	66 ± 17	58 ± 31
AUC (µM·3 h)	615,410 ± 42,914	720,015 ± 59,992 ^a^	672,951 ± 31,073 ^a^
***Insulin***			
C_max_ (µM)	30 ± 20	64 ± 43 ^a^	62 ± 43^a^
T_max_ (min)	34 ± 14	41 ± 14	36 ± 12
AUC (µM·3 h)	2697 ± 2229	4100 ± 2939 ^a^	4305 ± 3538 ^a^

^a^ Significantly different from NEAA; ^b^ significantly different from WPI; *Abbreviations:* AAs, total amino acids; AUC, area under the curve; BCAAs, branched-chain amino acids; BWPH, blue whiting protein hydrolysate; C_max_, peak concentration; EAAs, essential amino acids; NEAA(s), non-essential amino acid(s); T_max_, time to peak concentration; WPI, whey protein isolate.

## Data Availability

The data presented in this study are available in the results section and the supplementary Figure S1.

## Supplementary Materials

The following are available online at https://www.mdpi.com/2072-6643/13/2/647/s1, Individual amino acid concentrations following ingestion of non-essential amino acids (NEAA, ▲), whey protein isolate (WPI, ■), and blue whiting protein hydrolysate (BWPH, ○) in serum obtained from healthy older adults are shown in Figure S1. Data are presented as mean ± standard deviation.
