# Supplementary figures and images for: A Fish-Derived Protein Hydrolysate Induces Postprandial Aminoacidaemia and Skeletal Muscle Anabolism in an In Vitro Cell Model Using Ex Vivo Human Serum
Source: Nutrients. 2021 Feb 17;13(2):647. doi: 10.3390/nu13020647 (PMC7922518; doi:10.3390/nu13020647)

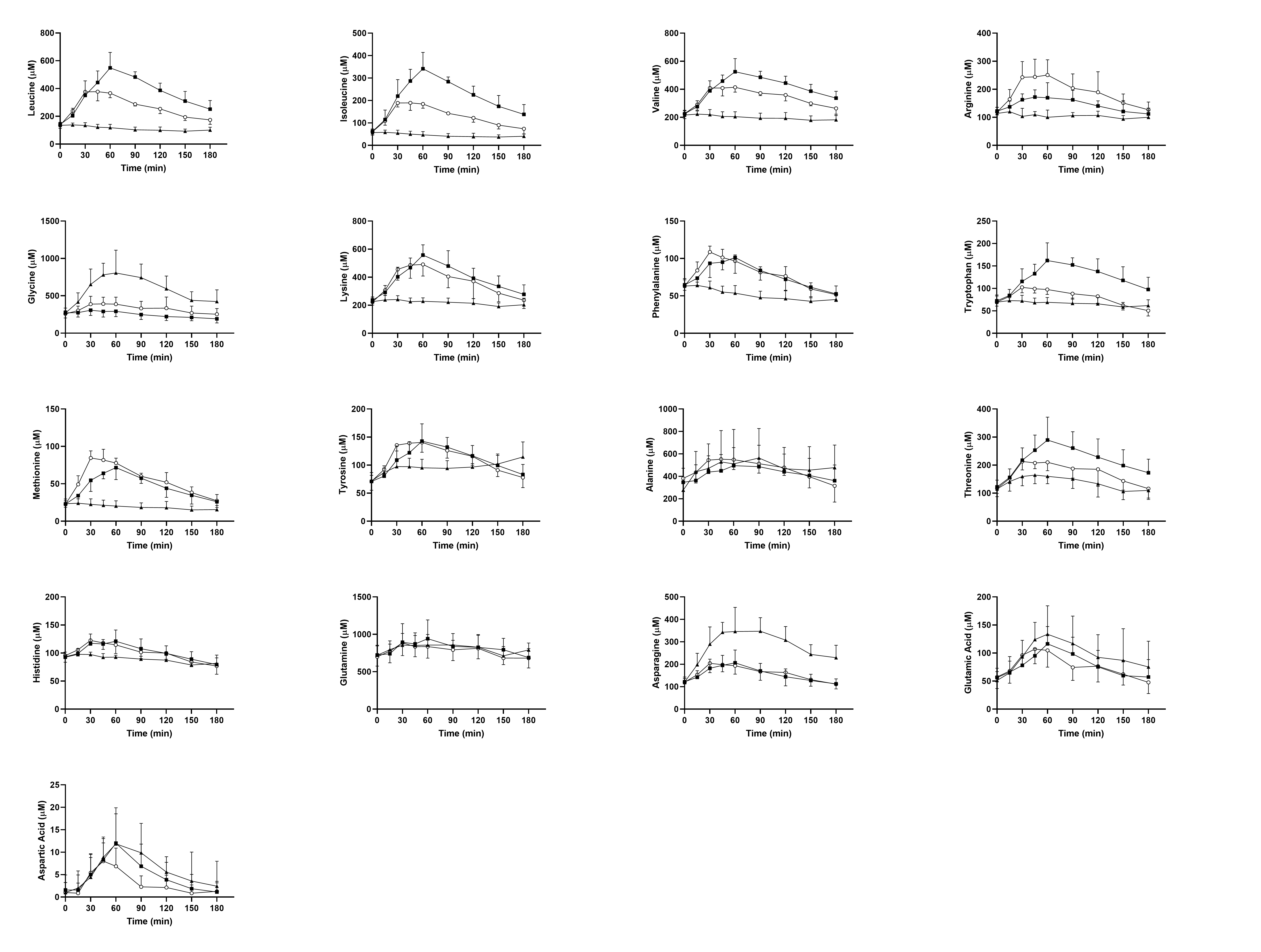

Supplement: Supplementary file 1 [file nutrients-13-00647-s001.zip › nutrients-1106797-supplementary.tif]
